# Complete Dislocation of the Lower Jaw Reduced by a New Method

**Published:** 1856-01

**Authors:** W. Colles


Complete Dislocation of the Lower Jaw reduced by a new method.—By W. Colles.

Miss ——, æt. 25, whilst indulging in a protracted yawn, felt a jerk in the jaw, and found she could not close her mouth. She immediately applied to a medical man in her neighborhood, who recognized a dislocation, but failed in his efforts at reduction. She then applied to Mr. Colles. " The mouth was open, the symphysis of the jaws slightly projecting. On applying the fingers to the angle of the jaw, and tracing the ramus upward, it led in a direction much anterior to the position of the articulating surface. The posterior edge of the bone could be most distinctly felt, and a broad furrow or hollow existed between the bone and the ear. The heads of the bone were felt and perceived prominent in front of their natural position, so that the face appeared broader at this place than natural.

"Before attempting reduction, I wished to ascertain the position in which I would have most command of the force to be used. Standing before her, I passed both thumbs into the mouth, but felt I would not have a position the most favorable for applying all my force, if necessary.

"I then stood behind her, and it at once struck me this was the position which afforded most advantages.

"Placing her head against my chest, I passed each thumb as far back on the corresponding side of the jaw as possible. By making a rotatory motion from the wrist, I found the bone to yield; by now adding a motion of drawing the hand in towards the chest, the left side first, then the right, slipped into their positions, and the patient closed the mouth, the rows of teeth falling into their relative positions, and she now could speak plainly.

"I think there are many advantages to be derived from attempting reduction in this posture, viz: the surgeon standing behind the patient, the head applied to his breast, and the thumbs turned inwards on the corresponding angles of the jaw, the fingers under the bone in front.

"In the first place, the head is much more secure than in the original process, where it is applied against a wall, because in the latter the surgeon may press down the bone, and the patient generally will draw the head in the same direction by moving the body forward in the chair.

"By standing behind the patient, while depressing and pushing back the thumbs, he is pressing forward with the chest, and thus fixes the head more steadily, and assists his manipulations; and even if the patient do move on the chair, a slight motion of his body will suffice to counteract this movement, and retain the head steadily fixed.

"Another advantage is, that he can use much more force, because when standing in front he can only use the muscles that depress the hands; whereas standing behind the patient he has the power of those muscles, and is assisted by the powerful class of muscles that rotate the thumbs inwardly; and, besides, in the former case his pressure is away from his body, whereas in the new position the pressure is more directly downwards and towards himself. The only disadvantage in this proceeding, if it can be considered one, is, that the mouth is stretched more than in the original plan."—*Dublin Hosp. Gaz.*, July 15, 1855.

